# Primary Echinococcus Hydatid Cyst of the Uterus: An Unusual Location

**DOI:** 10.1155/2021/9977326

**Published:** 2021-05-21

**Authors:** Farouk Ennaceur, Dhekra Toumi, Farouk Jaouad, Aymen Mabrouk, Ahmed Hajji, Mouna Gara, Atef Chamakh, Ines Zouari, Mohamed Maatouk, Sami Daldoul, Sofien Sayari, Karim Haouet, Yacine Ben Safta, Raja Faleh, Mounir Ben Moussa

**Affiliations:** ^1^Department of General and Digestive Surgery A at the University Hospital Charles Nicolle, University of Tunis el Manar, Tunisia; ^2^Department of Gynecology and Obstetrics at the University Hospital of Monastir, University of Monastir, Tunisia; ^3^Department of Anesthesiology at the University Hospital of Monastir, University of Monastir, Tunisia

## Abstract

Hydatidosis is a widespread anthropozoonosis. It can affect almost any part of the body, but it occurs most commonly in the liver (75%) and the lungs (15%). Its occurrence in female genital tract, especially the uterus, is very rare. Diagnosing hydatid disease at these unusual locations can be difficult. Hereby, we report two cases of primary hydatid cyst of the uterus. The first case is that of a 62-year-old woman, G7P5A2, who presented with an eight-month history of chronic pelvic pain. Clinical examination and radiological explorations revealed the presence of a uterine fibroid and a serous cystadenoma of the left ovary. She underwent a hysterectomy and a bilateral adnexectomy. Anatomopathological examination concluded that a serous cystadenoma of the left ovary was a calcified subserous hydatid cyst of the uterine fundus. The second case is that of a 69-year-old woman, G6P4A2, who consulted for chronic pelvic pain that had been evolving for 3 months. The clinical examination and radiological explorations doubted a hydatid cyst of the uterus, with a positive hydatid serology. She underwent a resection of the salient dome. The anatomopathological examination was in favor of a hydatid cyst of the uterus. Hydatid disease is endemic in Tunisia. The pelvic region is rarely affected with an incidence ranging from 0.3 to 0.9%, 80% of which involves the genitals. The uterus is more rarely affected than the ovaries. Most often, it is a contamination secondary to the intra-abdominal rupture of a hydatid cyst of the liver. However, primary uterine hydatid cysts have been reported. Surgery is the Gold Standard for the treatment of uterine hydatid cysts. Exploration of the abdominal cavity is essential in the search for other localizations, particularly hepatic. Postoperative medical treatment with Albendazole can be discussed. The ideal approach to deal with this public health concern is to emphasize the need for improved preventive measures. Modern imaging techniques have significantly improved the detection rates of hydatid cysts in atypical localizations. Indeed, the preoperative diagnosis of uterine hydatidosis requires a meticulous approach which is necessary to initiate an adequate treatment and thus guarantee a better management of the patient.

## 1. Introduction

Hydatidosis is a widespread anthropozoonosis worldwide. It is a real public health concern in countries where it is endemic. This zoonosis is caused by the larval form of a cestode of the Echinococcus granulosus species, a dog tapeworm for which man is an accidental intermediate host [1]. Hydatid disease can affect almost any part of the body except the nails, hair, and cornea. The most affected organs are the liver (75%) and the lungs (15%) [2]. The involvement of female genital tract, especially the uterus, in hydatid disease is extremely rare. The diagnosis of these unusual forms requires a thorough history taking, a detailed clinical examination, serological tests, and various imaging techniques (ultrasound, CT scan, and MRI). Hence, the importance of this article reports 2 cases of primary hydatid cyst of the uterus [3, 4].

## 2. Observations

### 2.1. Observation 1

This is a 62-year-old woman, menopausal for 12 years, hypertensive, G7P5A2, with no previous history of abdominal surgery, who consulted for chronic pelvic pain evolving for 8 months.

**Figure 1:**
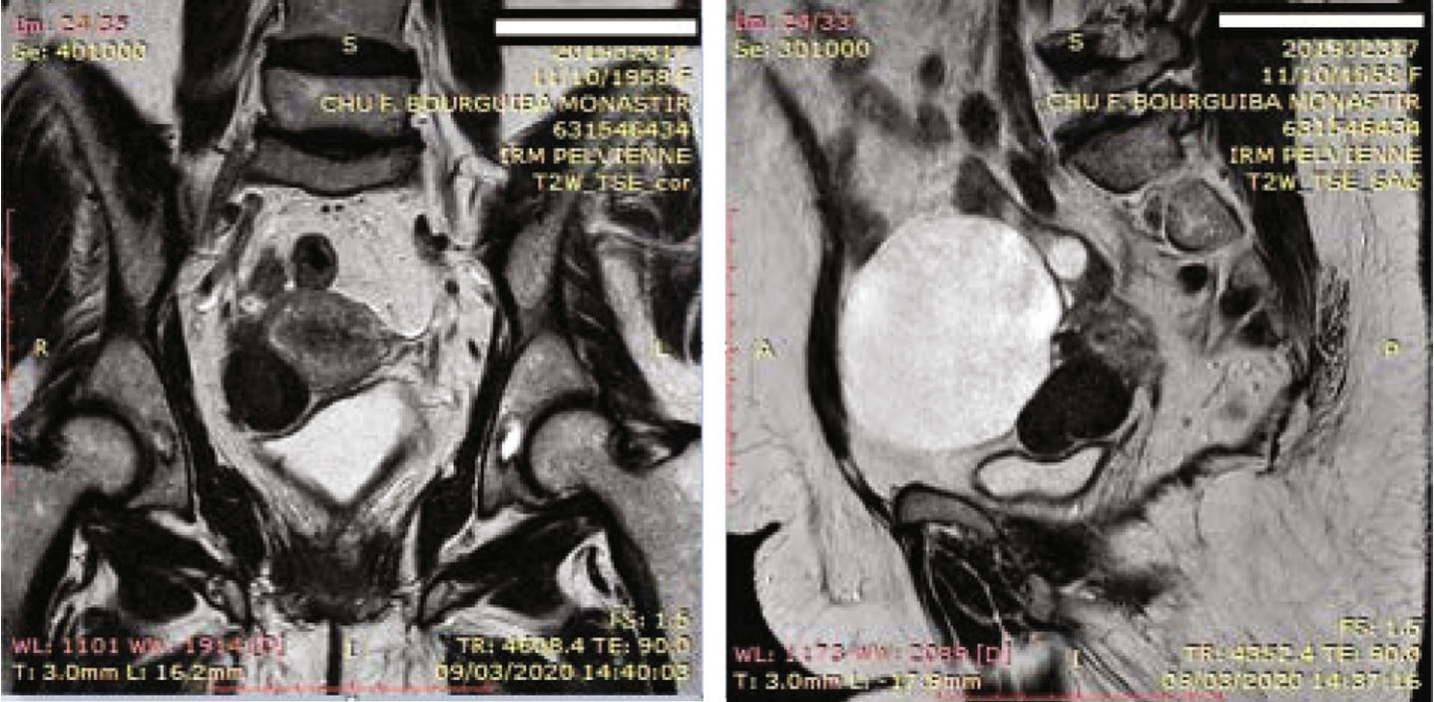
MRI aspect of the cyst.

Abdominal examination did not reveal any abnormalities. Vaginal speculum examination revealed a macroscopically healthy cervix, a 1 cm polyp of the cervix. Vaginal touch revealed a small uterus, with the perception of a renitent left latero-uterine mass separated from the uterus by a groove. A pelvic ultrasound revealed a 4 cm uterine-fundal hypoechoic mass evoking a uterine fibroid. The left ovary was the site of a cystic mass measuring 10 cm in diameter with pure anechogenic content. A pelvic MRI scan was performed and concluded a 9 cm purely cystic mass of the left ovary classified as O-RADS 2 evocative of a serous cystadenoma, with a pedicle subserous FIGO type 7 myoma developed at the expense of the anterior wall of the uterine body lateralized on the right measuring 30∗27∗40 mm (frank T2 hyposignal without diffusion restriction) (Figure 1).

The patient was operated on, and an exploration of the abdominal cavity revealed a uterus was surmounted by a 4 cm cystic lesion with subserous development and a 12 cm cystic mass with no exocystic vegetations in the left ovary. Given the patient's age, the diagnosis of a malignant lesion has been evocated, so a total hysterectomy with bilateral adnexectomy was performed. The postoperative follow-up was unremarkable. The patient was discharged four days postoperatively.

**Figure 2:**
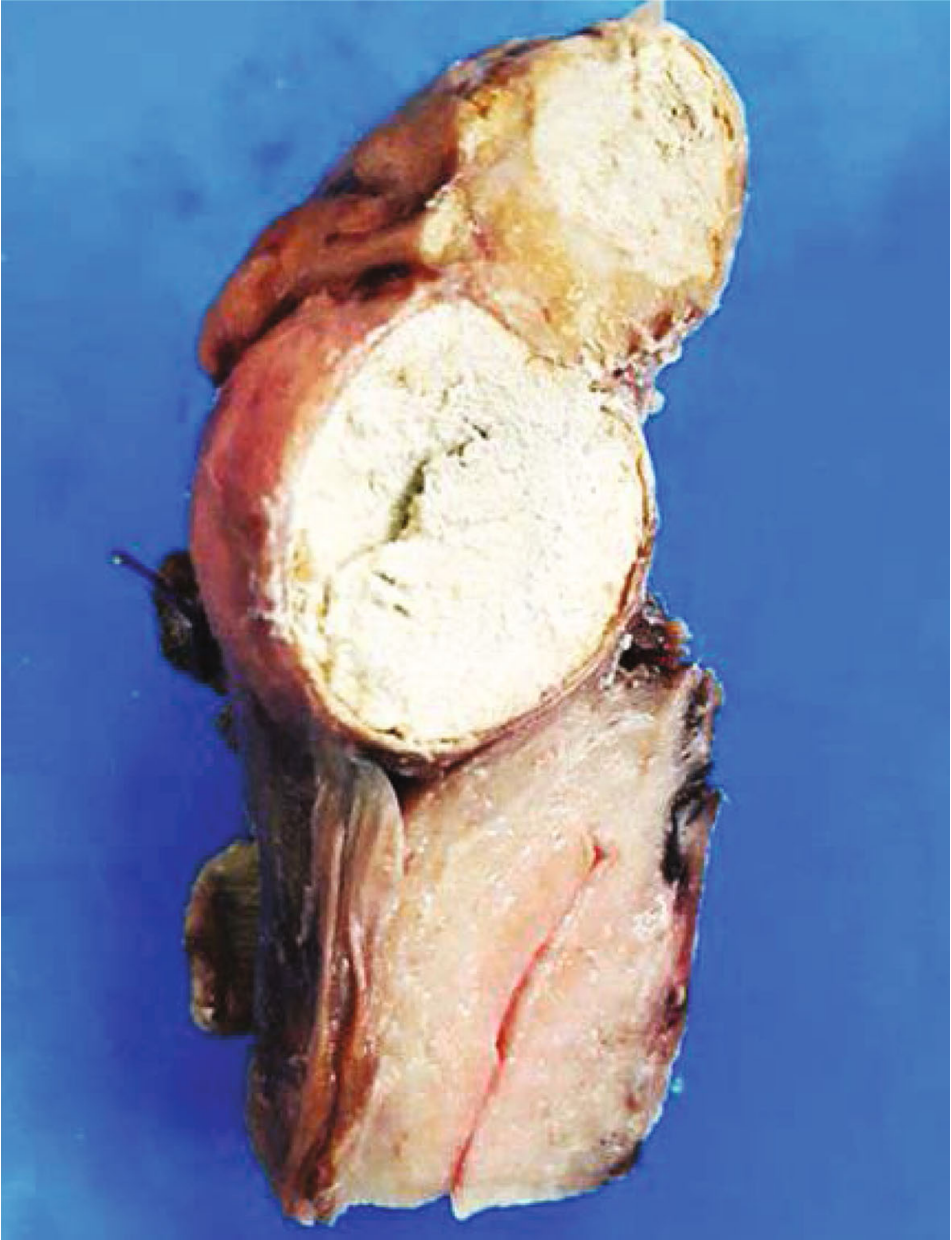
Macroscopic aspect of the cyst.

Anatomopathological examination concluded two ovarian serous cystadenomas measuring 12 cm on the left and 1.7 cm on the right and a subserous calcified hydatid cyst of the uterine fundus with a benign polyp of the endocervix (Figure 2).

A chest X-ray and an abdominal ultrasound were subsequently performed and did not reveal any other hydatid localizations.

### 2.2. Observation 2

This is a 69-year-old overweight woman (BMI = 27 kg/m^2^), G6P4A2, menopausal for 16 years, hypertensive, and diabetic, with no previous history of abdominal surgery, who consulted for chronic pelvic pain evolving for 3 months.

**Figure 3:**
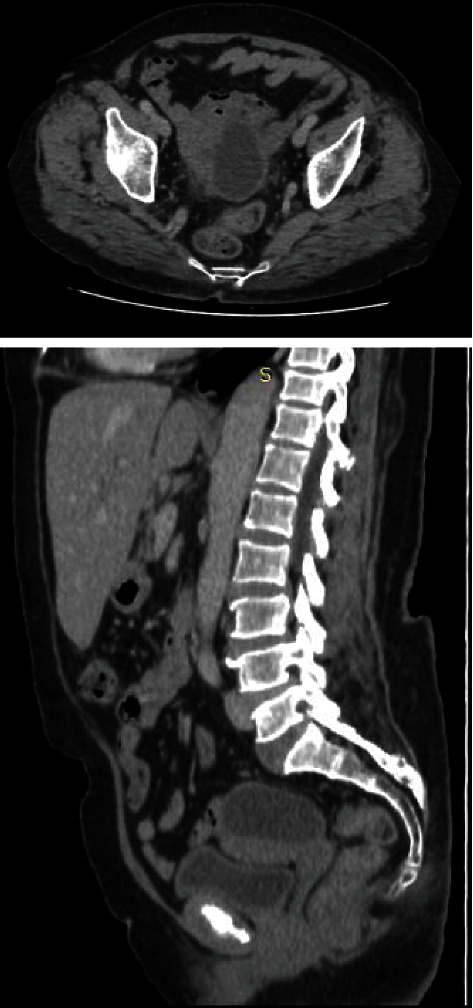
CT aspect of the cyst.

Physical examination revealed a painless abdomen without palpable mass. Vaginal speculum examination showed a macroscopically healthy cervix. On vaginal touch, there was a painless small uterus. The rest of the clinical examination was without particularities. A pelvic ultrasound scan was performed and showed a 5 cm mass in the uterine fundus. An additional scan substantiated the presence of a 5 cm hydatid cyst in the uterine fundus, with no other localizations detected (Figure 3).

Chest X-ray did not objectify the lesions. The hydatid serology was positive (1/320) according to the ELISA technique (enzyme-linked immunosorbent assay). The patient was operated on medially, a hydatid cyst of the uterine fundus was found, but no hepatic lesions were noticed, and the protruding dome was resected (we opted for a conservative treatment since we operated the patient with a preoperative diagnosis of a benign cyst). The operation was uneventful, and the patient was discharged 4 days after the operation. The anatomopathological examination was in favor of a hydatid cyst of the uterus.

## 3. Discussion

Hydatid disease is endemic in Tunisia; it is concentrated in the northwest of the country. Globally, the endemic areas are the Mediterranean countries, the Middle East, the southern part of South America, Iceland, Australia, New Zealand, and southern parts of Africa; the latter five regions are intensive endemic areas. Central Asia, particularly China, is also an endemic area. Approximately 2 to 3 million cases of human echinococcosis are reported worldwide each year [5].

Echinococcus granulosus induces a cystic, noninvasive, unilocular, and encapsulated lesion that evolves slowly (0.5 to 3 cm per year). It commonly affects only one organ, and 10 to 15% of cases have two organs affected [6].

The pelvic region is rarely involved with an incidence ranging from 0.3 to 0.9%, of which 80% involve the genitals. The uterus is more rarely affected than the ovaries [7]. The majority of cases were reported in the literature concern patients aged between 20 and 40 years. Contamination is most often secondary to intra-abdominal rupture of a hydatid cyst of the liver; the daughter vesicles and scolices released settle in the cul-de-sac and continue to develop; secondary endothelialization excludes them from the peritoneal cavity; thus, the intraperitoneal cyst becomes extraperitoneal and appears to be part of the uterine cell tissue. However, primary uterine hydatid cysts have been reported [8].

The rarity of uterine hydatidosis, the slow progression of this disease, and the heterogeneity of symptoms make preoperative diagnosis difficult. Differential diagnosis is made with cystic or mixed retroperitoneal tumors (dermoid cysts), pyogenic or tuberculous abscesses, cysts of the ovary or hydrosalpinx, ovarian tumors, or uterine fibroids [9].

Surgery is the Gold Standard for the treatment of uterine hydatid cysts. Midline laparotomy under the umbilicus remains the best choice. The laparoscopic approach remains controversial because of the lack of experience in treating hydatid cysts by this route, the increased risk of dissemination, and the higher rate of recurrence. Sterilization of the cyst and protection of the surgical site are performed using hydrogen peroxide. A cystectomy must be performed for accessible cysts or for the partial or total surgical removal of organs invaded by hydatidosis [10]. Exploration of the abdominal cavity is essential in the search for other localizations, particularly hepatic. Medical treatment is not very effective and is used in the case of operative contraindications or multiple localizations or if the resection is incomplete. This medication is based on Albendazole (400 mg/day; two 28-day cures 15 days apart) [11].

In an endemic country such as ours where hydatidosis is a public health concern, the ideal is to focus on the implementation of preventive measures, notably the fight against infestation of the definitive host, the protection of the intermediate host, and the fight against human contamination.

## 4. Conclusion

The detection of hydatid cyst in its atypical localizations has been facilitated by various modern imaging techniques. Indeed, the preoperative diagnosis of uterine hydatidosis requires a meticulous approach which is necessary to initiate an adequate treatment and thus guarantee a better management of the patient.
